# Effects of nighttime odor exposure and delivery methods on subjective sleep quality in healthy adults

**DOI:** 10.1038/s41598-025-18075-x

**Published:** 2025-09-12

**Authors:** Zetian Li, Jean Christoph Gerami-Manesch, Lisa Marie Hoehnel, Jonathan Warr, Antje Haehner, Thomas Hummel

**Affiliations:** 1 Department of Otorhinolaryngology, Faculty of Medicine Carl Gustav Carus, Smell and Taste Clinic, Technische Universität, Fetscherstraße 74, 01307 Dresden, Germany; 2 Takasago Europe, Paris, France

**Keywords:** Application, Diffuser, Fitbit, Olfaction, Sleep, Human behaviour, Sleep disorders

## Abstract

The role of smell in improving sleep quality is inconclusive. This study aimed to investigate whether the type of odor and the method of application affect sleep quality. Based on individual preference 112 healthy participants (mean age ± SD = 31 ± 12 years, 43 men) selected an odor (lavender, orange, specifically designed “perfume”) and an application method (odorized nasal clips, odors on pillow, odors through diffuser). Their performance was compared to a control group (*n* = 19; mean age ± SD = 29 ± 6 years, 9 men). Except for the control group, all groups slept with exposure of odor for 2 weeks (phase 1), followed by 2 more weeks of normal sleep without odors (phase 2). The Pittsburgh Sleep Quality Index (PSQI) and the wearable sleep monitoring device “Fitbit Charge 2” were used to measure sleep quality both subjectively and objectively. After adjusting for baseline differences, participants exposed to “perfume” reported better subjective sleep quality during the odor exposure phase compared to the control group. Among the application evaluated, the use of a diffuser was associated with better subjective sleep quality and reduced daytime fatigue, as reflected in the PSQI components. No significant group differences were observed in objective sleep parameters measured by Fitbit. Exposure to odors during the night influence sleep quality. Using a diffuser to deliver the odor appears to be associated with better subjective sleep quality and reduced daytime fatigue. No sustained improvement in sleep quality over time was observed with odor exposure.

**Supplementary Information:**

The online version contains supplementary material available at 10.1038/s41598-025-18075-x.

## Introduction

Olfaction, as an implicit sensation, offers a unique pathway to impact sleep patterns without leading to behavioral or physiological arousal^1,2^. The literature shows that olfaction modulates the neural process during sleep^3,4^, and impacts both dreams and sleep quality^5–7^. A recent study of COVID-19 survivors suggests those who lost their sense of smell were more likely to have poor sleep quality, further implicating the close association between sleep and olfaction^8^.

Exposure to odors has been found to facilitate sleep quality. Certain fragrant substances, such as lavender, are anecdotally believed to enhance sleep quality. A scientific review supports this claim, suggesting small to moderate benefits^9^. Aromatherapy, which utilizes the smell of essential oils, is thought to promote sleep quality^10^. However, most sleep outcomes in these studies were based on self-ratings of participants and involved small sample sizes^10,11^.

A recent study involving 139 participants indicated that the hedonic value of the odor to the participant, rather than the odors per se, appears to be crucial for sleep quality^12^. This study found no consistent improvement in sleep quality for a specific odor based on data from subjective ratings and wearable sleep monitoring devices. The authors suggested that this inconsistency may be due to the method of odor exposure via nasal clips, which did not accurately replicate the ambient atmosphere in which odors are typically encountered in home settings.

Hence, the aims of the present study were to further investigate whether exposure to certain odors would improve subjectively or objectively measured sleep quality, exploring alternative means of introducing the odor. More specifically it was of interest to investigate whether the method of direct (e.g., using nasal clips, or odors applied to the pillow) or indirect (e.g., using a diffuser, distributing odors in the room) odor presentation during sleep affect sleep quality.

## Methods and materials

### Sample

A total of 131 healthy participants were recruited between June 2022 and September 2023. Of these, 112 participants (mean age ± SD = 31 ± 12 years, 43 men) selected an odor (lavender, orange, or a specially designed “perfume”) and an application method (nasal clips, pillow, or diffuser) for nighttime odor exposure during sleep. An additional control group of 19 participants (mean age ± SD = 29 ± 6 years, 9 men) was also included. The inclusion criteria were: (1). Voluntary participation; (2). Age of at least 18 years; (3). Normal sense of smell. Exclusion criteria were: (1). Pregnancy and breastfeeding; (2). Tobacco dependence (more than 5 cigarettes per week); (3). Acute or chronic inflammation of the nose and sinuses; (4). Irregular sleep patterns (e.g., shift work, caring for an infant); (5). Illnesses affecting olfactory function. The present study was approved by the Ethics committee at the Medical Faculty Carl Gustav Carus of the Technische Universität Dresden (application number EK377082019). All participants gave written informed consent.

### Measures

The odors of lavender oil (Wesentlich, Willich, Germany; article number WES20080), orange oil (Elixens, France; article number 015026), and specifically designed “perfume” (Takasago, Paris, France; article number BLE312740) were selected, using the same formulation as in our previous study^12^. Description of the odors are listed in Table 1. Based on a pilot study, each odor was diluted in solvent to achieve a comparable level of intensity amongst all groups: for the nasal clips and pillow spray dipropylene glycol was used and for the diffuser Dowanol DPM (details see Appendix 1). Since the individual pleasantness of the odor seems to influence sleep quality^12^, participants were divided into four different groups according to their preference for odors: (1). lavender, *n* = 37, 11 men, mean age ± SD = 32 ± 12; (2). orange, *n* = 37, 15 men, mean age ± SD = 33 ± 12; (3). “perfume”, *n* = 38, 17 men, mean age ± SD = 29 ± 10; (4). control, *n* = 19, 9 men, mean age ± SD = 29 ± 6;

**Table 1:** Description of odors used in the study.

Odor	Description
Lavender	Essential lavender oil extracted by steam distillation of Lavandula angustifolia plant matter (stem, leaves, flowers).
Orange	Essential oil obtained by scraping the fresh pericarp of Citrus sinensis.
Specifically designed “perfume”	Floral-musk scent with top notes of citrus and orange, middle notes of white flowers, rose, and jasmine, and base notes of violet, orris, and musk, primarily composed of hedione, musk T, ionone beta, linalool, and minor citrus elements.

Three application formats were used to present the smell: nasal clips, pillow spray, and reed diffuser (details see Appendix 1). Disposable nasal clips, a small, clear, soft plastic nasal clip in the shape of a horseshoe, approximately 2 cm in size (manufactured by aspUraclip, Berlin, Germany), allow specific odor presentation to an individual without contaminating the environment^12,13^. Pillow application was employed by dispersing odor on the pillow.

Diffusers, consisting of brown jars (50 ml volume; neoLab Migge GmbH, Heidelberg, Germany) containing the odorous solutions and three reed sticks to emanate the scents, were placed near the participants’ beds. Four groups based on the application were: (1). clip, *n* = 32, 14 men, mean age ± SD = 33 ± 12; (2). pillow, *n* = 41, 13 men, mean age ± SD = 30 ± 11; (3). diffuser, *n* = 39, 28 men, mean age ± SD = 31 ± 11; (4). control, *n* = 19, 9 men, mean age ± SD = 29 ± 6.

Participants were distributed as follows: lavender group (9 clip, 15 pillow, 13 diffuser), orange group (11 clip, 11 pillow, 15 diffuser), and “perfume” group (12 clip, 15 pillow, 11 diffuser).

Olfactory function was measured by the Sniffin’ Stick test (SST), which is based on sets of reusable pen-like odor dispensers^14^. SST comprises three subtests: odor threshold, identification, and discrimination. The combined Threshold-Discrimination-Identification (TDI) score is then calculated as the sum of the individual scores from the odor threshold, discrimination, and identification tests, ranging from 1 to 48. A higher TDI score indicates better olfactory function.

The Pittsburgh Sleep Quality Index (PSQI) is a self-rated questionnaire, designed for assessing sleep quality^15^. Nineteen items generate seven component scores, which include subjective sleep quality, sleep latency, sleep duration, habitual sleep efficiency, sleep disturbances, use of sleeping medication, and daytime dysfunction. The sum of the seven component scores yields the total score of PSQI, ranging from 0 to 21, where the higher the score, the worse the sleep quality.

Fitbit Charge 2 (Fitbit Inc., San Francisco, CA, USA) is a wrist wearable sleep monitoring device, which could measure the total sleep time, waking time, time in bed, time for light sleep, deep sleep, and rapid eye movement (REM) sleep^16^.

### Procedure

**Fig. 1:**
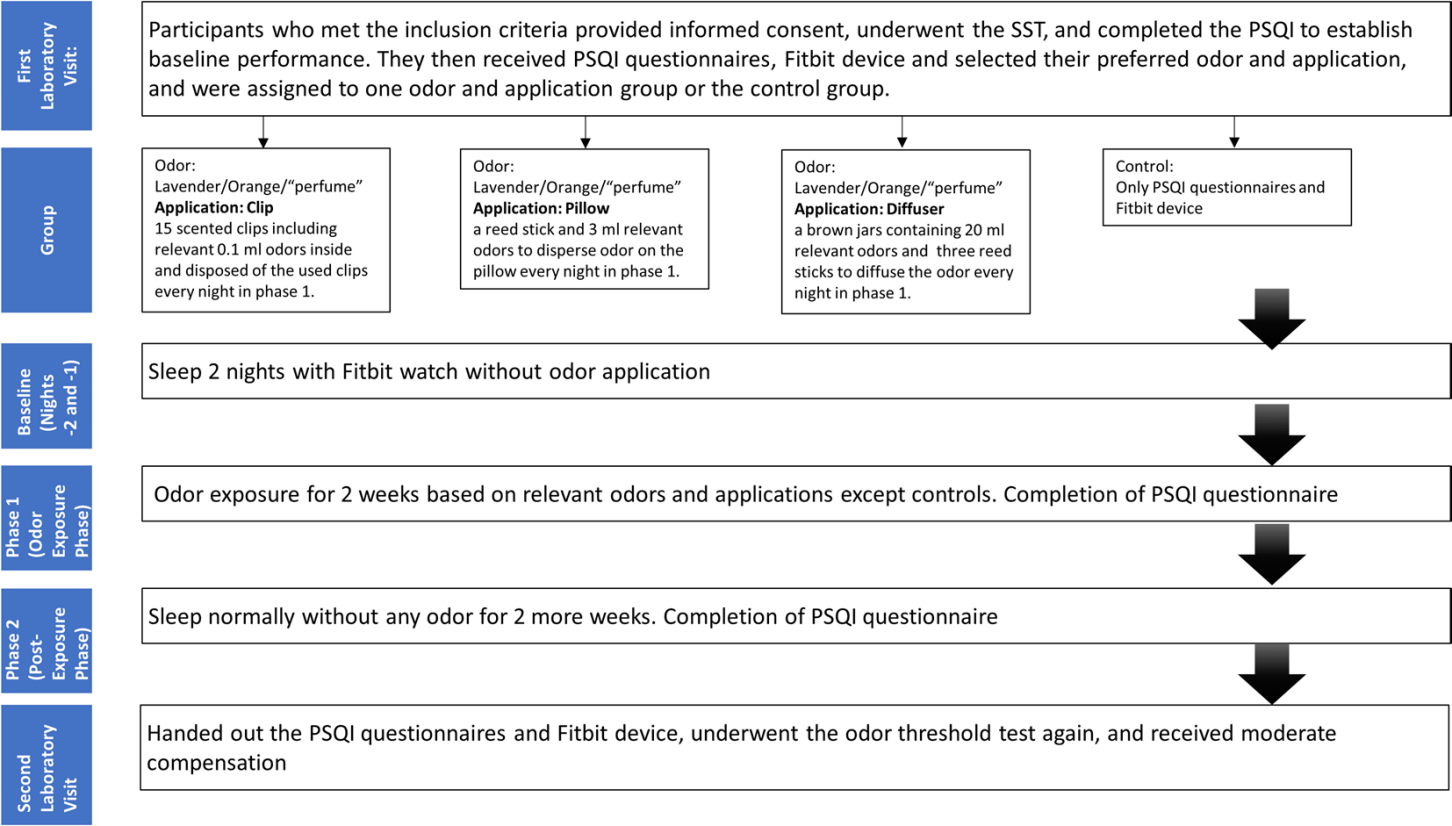
Flow chart of the experiment.

Figure 1 shows the procedure of the experiment. Before recruitment, participants underwent pre-selection according to the inclusion and exclusion criteria. Upon visiting the laboratory all participants provided informed consent, underwent the SST, completed the PSQI to establish baseline performance, selected their preferred odor and application, and received essential documents and materials (questionnaires for home, odor, application and Fitbit device) during the initial appointment. Participants in the clip group received 15 scented clips including approximately 0.1 ml odor inside and disposed of the used clips every night. Those in the pillow group were required to disperse odor on the pillow using a reed stick, with a distinctly perceptible scent intensity at the beginning of the night. Brown jars (50 ml) volume containing 20 ml odor were distributed to participants in the diffuser group. The control group slept with no odor exposure nor any applications environment throughout the experiment. Participants also received a daily sleep diary throughout the study period, documenting behaviors such as their daily intake of coffee and alcohol.

All participants slept at home and received comprehensive instructions covering all aspects of the study, such as how to properly apply the odor before bedtime. During the first two nights, all participants slept without any odor exposure while wearing a Fitbit watch to establish a baseline, followed by two experimental phases. In phase 1, all participants, except those in the control group, were exposed to the selected odor during sleep for the initial two-week period. In phase 2, participants slept two more weeks without any odor exposure. Participants were required to complete PSQI after both phases. During the second laboratory visit, participants were handed out the questionnaires, underwent only the odor threshold test again, due to time limitations, in order to explore potential changes in peripheral olfactory sensitivity following nighttime odor exposure, and received moderate compensation for their participation.

### Data analysis

Data analysis was performed using SPSS (version 29.0; Armonk, NY, USA). Univariate analysis of variance (ANOVA) was used to analyze age and TDI total scores across different odor and application groups, while chi-square tests assessed gender ratios. To examine differences following odor exposure, univariate ANCOVA models included baseline scores as covariates and compared PSQI total and subcomponent scores in phase 1, analyzed separately by odor type and application method, with Bonferroni corrections for post hoc comparisons. Similar ANCOVA models assessed Fitbit-derived sleep outcomes, including total sleep time, wake time, time in bed, and durations of light, deep, and REM sleep. Additionally, linear mixed multivariable models were used to evaluate the effects of session (baseline, phase 1, phase 2), odor (lavender, orange, “perfume”, control), and application (clip, pillow, diffuser, control) on PSQI and Fitbit outcomes, followed by Bonferroni-corrected post hoc tests. Finally, a linear mixed model was also used to analyze changes in odor threshold between the two lab visits. Two-tailed *P* < 0.05 denoted significance.

## Results

### Demographic and olfactory information

Descriptive data are shown in Tables 2 and 3. Ages of participants were comparable between all groups (odor: *F* [3,130] = 1.05, *p* = 0.38, *η*^2^ = 0.024, application: odor: *F* [3,130] = 0.49, *p* = 0.69, *η*^2^ = 0.011), and their gender ratio was not significantly different between groups (odor: *χ*^*2*^ [6] = 4.67, *p* = 0.59, φ = 0.19; application: *χ*^*2*^ [6] = 4.25, *p* = 0.64, φ = 0.18). Based on the standard clinical cutoff (PSQI > 5^15^, , approximately 26% of participants at baseline were classified as having poor sleep quality, implying that some participants may have had compromised sleep quality at baseline. Participants had a normal sense of smell as ascertained with the SST (mean TDI total score ± SD: 34.23 ± 3.18^17^. There were no significant group differences in olfactory function, PSQI scores, or Fitbit data at baseline (all *p* > 0.05).

**Table 2:** Demographic information, olfactory function and sleep measurement results for the odor of lavender, orange, specifically designed “perfume” and control groups.

	Lavender (*n* = 37)	Orange (*n* = 37)	“Perfume” (*n* = 38)	Control (*n* = 19)
Age (years)	32.11 ± 12.40	33.16 ± 11.75	29.26 ± 10.23	29.47 ± 4.18
Gender ratio (M: W)	12:25	15:22	17:21	9:10
Sniffin’ sticks test				
Total score	34.80 ± 3.54	34.60 ± 2.74	33.20 ± 3.09	34.45 ± 3.10
1st Threshold	8.64 ± 2.53	7.95 ± 2.33	7.30 ± 2.28	7.76 ± 2.39
2nd Threshold	8.37 ± 2.69	8.29 ± 3.15	8.81 ± 2.82	8.80 ± 1.83
Baseline (without odor)				
PSQI total	3.43 ± 1.63	4.16 ± 2.15	3.97 ± 1.08	3.74 ± 1.73
Fitbit data (mins)				
Total sleep time	410.59 ± 65.80	405.38 ± 63.28	428.28 ± 57.60	419.33 ± 46.74
Wake time	53.0 ± 14.22	62.48 ± 15.57	60.05 ± 18.15	59.42 ± 20.45
Time in bed	463.59 ± 73.85	472.44 ± 65.09	488.33 ± 70.56	478.75 ± 60.01
REM sleep	81.17 ± 26.20	87.50 ± 25.99	95.72 ± 26.89	86.33 ± 33.68
Light sleep	264.20 ± 53.49	243.98 ± 52.74	262.78 ± 41.13	262.42 ± 25.60
Deep sleep	65.22 ± 24.83	70.06 ± 30.45	70.58 ± 24.84	68.67 ± 27.01
Phase 1 (with odor)				
PSQI total	4.88 ± 2.34	4.81 ± 1.95	4.42 ± 1.27	4.74 ± 2.05
Fitbit data (mins)				
Total Sleep time	420.71 ± 48.14	404.38 ± 42.00	413.92 ± 39.48	411.70 ± 46.58
Waking time	61.61 ± 10.76	58.72 ± 10.79	58.32 ± 8.95	59.23 ± 11.55
Time in bed	482.36 ± 54.58	463.11 ± 48.42	472.25 ± 45.14	471.12 ± 54.90
REM sleep	79.15 ± 20.19	84.83 ± 19.98	83.60 ± 21.41	80.40 ± 19.63
Light sleep	271.80 ± 35.39	251.19 ± 25.27	261.77 ± 34.47	265.53 ± 36.96
Deep sleep	69.77 ± 17.03	68.36 ± 17.83	69.23 ± 13.84	65.81 ± 12.73
Phase 2 (without odor)				
PSQI total	5.00 ± 1.91	4.69 ± 1.91	4.76 ± 1.59	4.56 ± 1.95
Fitbit data (mins)				
Total sleep time	415.91 ± 47.57	414.57 ± 32.71	425.76 ± 42.56	409.37 ± 40.03
Waking time	58.00 ± 11.61	61.58 ± 15.96	60.60 ± 10.02	60.18 ± 13.04
Time in bed	473.92 ± 52.86	476.15 ± 39.49	486.36 ± 50.18	469.71 ± 47.96
REM sleep	79.74 ± 18.64	89.52 ± 18.55	87.97 ± 19.63	82.22 ± 20.60
Light sleep	270.34 ± 41.27	258.86 ± 20.08	266.56 ± 38.37	257.74 ± 34.30
Deep sleep	67.08 ± 15.30	66.19 ± 17.76	71.15 ± 13.87	70.08 ± 16.70

Note: M = Men, W = Women, PSQI = Pittsburgh Sleep Quality Index.

**Table 3:** Demographic information, olfactory function and sleep measurement results for the application of clip, pillow, diffuser and control groups.

	Clip (*n* = 32)	Pillow (*n* = 41)	Diffuser (*n* = 39)	Control (*n* = 19)
Age (years)	33.03 ± 12.15	30.66 ± 11.23	31.10 ± 11.42	29.47 ± 4.18
Gender ratio (M:W)	14:18	13:28	16:23	9:10
Sniffin’ sticks test				
Total score	34.53 ± 2.64	34.56 ± 3.19	33.43 ± 3.59	34.45 ± 3.10
1st threshold	8.63 ± 1.80	8.11 ± 2.47	7.25 ± 2.75	7.76 ± 2.39
2rd threshold	9.47 ± 2.99	8.81 ± 2.56	7.36 ± 2.39	8.80 ± 1.83
Baseline (without odor)				
PSQI total	3.66 ± 1.54	3.80 ± 1.60	4.08 ± 1.90	3.74 ± 1.73
Fitbit data (mins)				
Total sleep time	417.70 ± 57.59	412.53 ± 59.63	417.02 ± 70.55	419.33 ± 46.74
Wake time	60.52 ± 19.10	58.69 ± 15.50	57.36 ± 15.70	59.42 ± 20.45
Time in bed	478.23 ± 70.98	475.06 ± 64.49	474.38 ± 77.15	478.75 ± 60.01
REM sleep	93.36 ± 28.06	87.13 ± 26.85	86.52 ± 25.95	86.33 ± 33.68
Light sleep	255.43 ± 43.19	251.73 ± 51.10	264.70 ± 52.65	262.42 ± 25.60
Deep sleep	68.91 ± 30.86	70.45 ± 25.59	65.80 ± 26.99	68.67 ± 27.01
Phase 1 (with odor)				
PSQI total	4.61 ± 1.36	5.40 ± 2.10	4.05 ± 1.82	4.74 ± 2.05
Fitbit data (mins)				
Total sleep time	409.85 ± 43.08	416.07 ± 47.01	412.40 ± 40.67	411.70 ± 46.58
Waking time	57.71 ± 9.94	60.56 ± 10.39	59.97 ± 10.29	59.23 ± 11.55
Time in bed	467.55 ± 49.19	476.67 ± 54.60	472.37 ± 45.35	471.12 ± 54.90
REM sleep	85.45 ± 19.67	82.29 ± 22.28	80.41 ± 19.43	80.40 ± 19.63
Light sleep	261.42 ± 33.94	262.05 ± 32.57	261.25 ± 33.15	265.53 ± 36.96
Deep sleep	63.79 ± 18.32	71.75 ± 14.28	70.75 ± 15.51	65.81 ± 12.73
Phase 2 (without odor)				
PSQI total	4.41 ± 1.56	5.26 ± 2.00	4.72 ± 2.26	4.56 ± 1.95
Fitbit data (mins)				
Total sleep time	419.57 ± 34.46	418.81 ± 47.44	418.18 ± 40.70	409.37 ± 40.03
Waking time	56.52 ± 8.32	60.47 ± 11.96	62.55 ± 15.78	60.18 ± 13.04
Time in bed	476.09 ± 37.91	479.28 ± 57.28	480.73 ± 45.02	469.71 ± 47.96
REM sleep	89.84 ± 20.26	86.59 ± 19.33	81.55 ± 17.99	82.22 ± 20.60
Light sleep	265.14 ± 29.61	263.98 ± 37.58	266.72 ± 35.92	257.74 ± 34.30
Deep sleep	66.02 ± 15.93	68.18 ± 16.18	69.91 ± 15.24	70.08 ± 16.70

Note: M = Men, W = Women, PSQI = Pittsburgh Sleep Quality Index.

### ANCOVA results

**Fig. 2:**
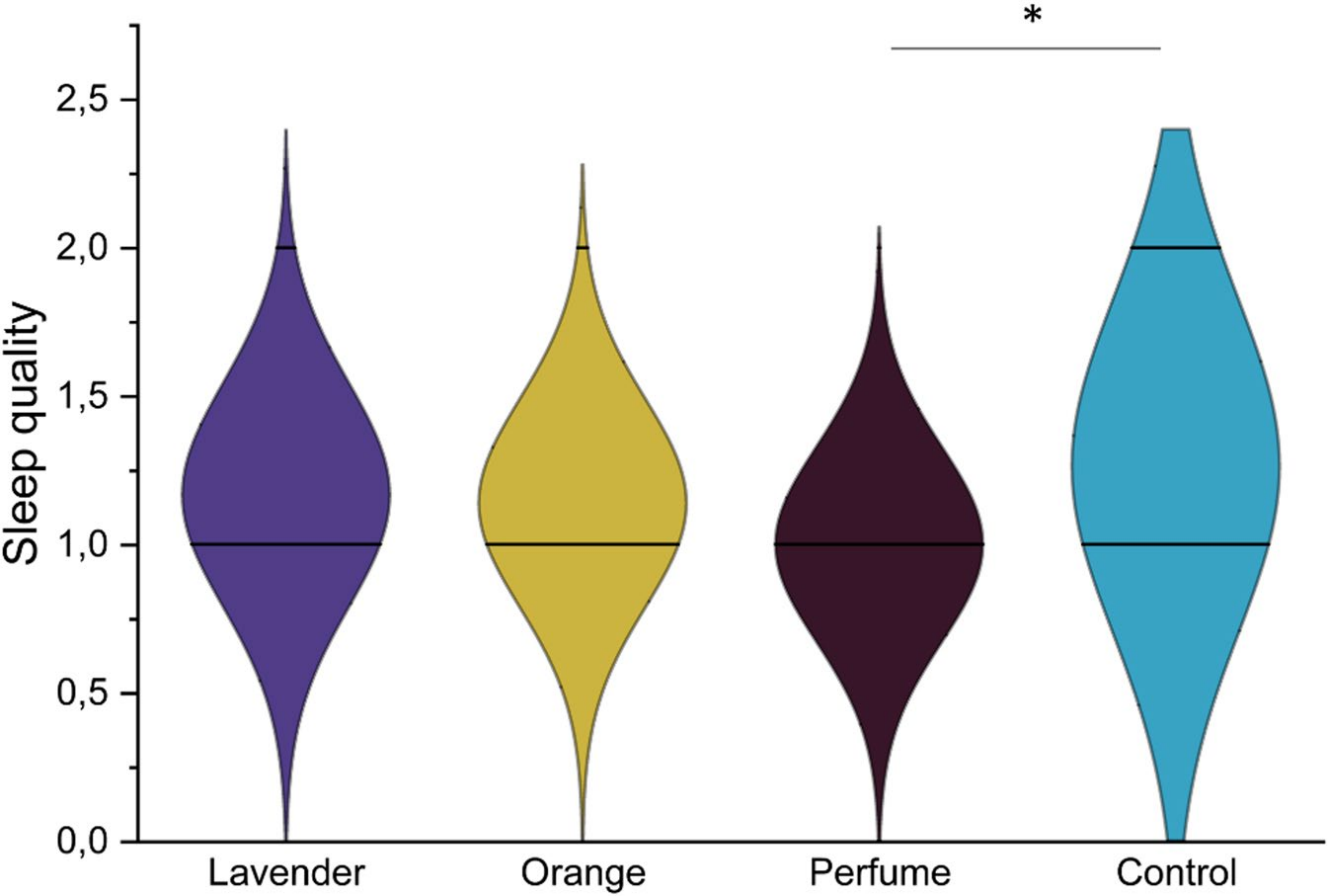
Ratings of subjective sleep quality (a component of Pittsburgh Sleep Quality Index) for different odor conditions (lavender, orange, “perfume”, and control). The higher the rating, the worse the sleep quality. After controlling baseline rating, participants using “perfume” had better sleep quality than the control group in phase 1 *p < 0.05.

To eliminate the baseline influence, the Univariate model ANCOVA analyses for phase 1 with odor exposure, with baseline performance as covariates were conducted. Due to missing data, two participants were excluded from the ANCOVA analyses. As the proportion of missing data was small, no data imputation was applied. For odor effect, a group difference was found in the subjective sleep quality (a component of PSQI, *F* [3,124] = 3.24, *p* = 0.024, partial η^2^ = 0.073, Fig. 2), with Bonferroni corrected pairwise comparison suggesting that after controlling baseline rating, participants using “perfume” had better sleep quality than the control group (*p* = 0.026). No differences were found in the PSQI Total score and other components (all *p* > 0.05).

**Fig. 3:**
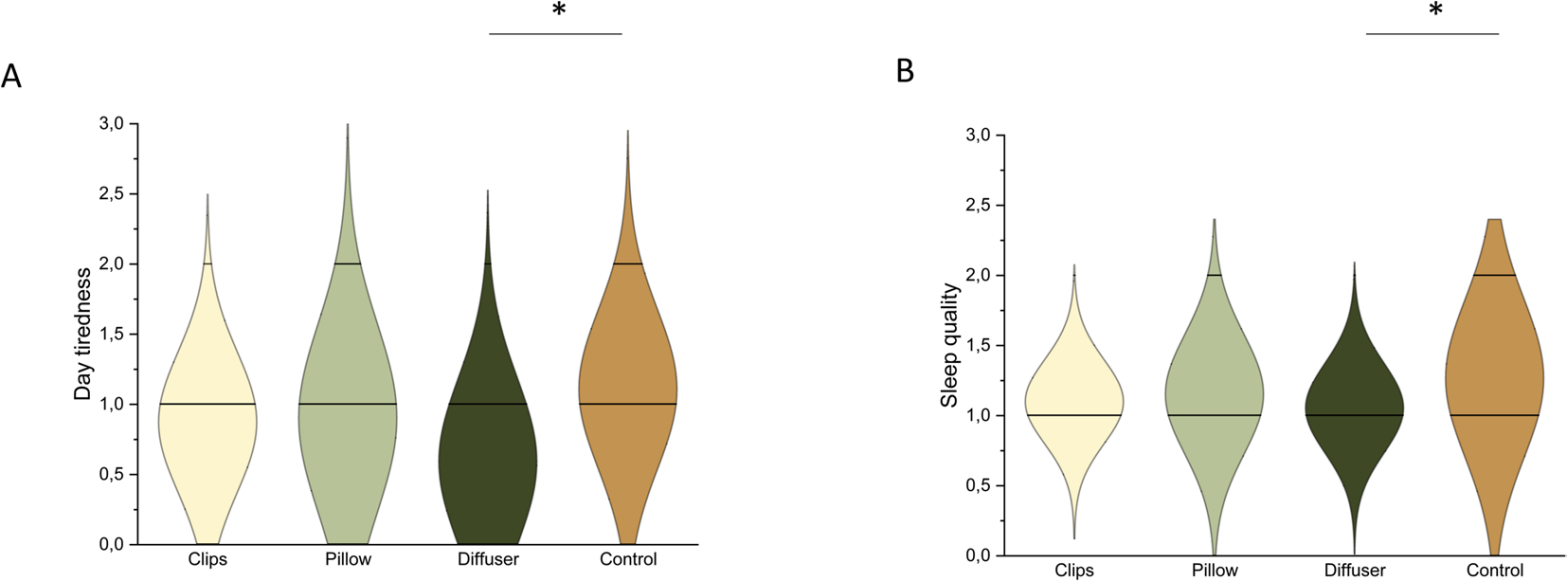
Ratings of (**A**) subjective sleep quality and (**B**) daytime tiredness (two components of the Pittsburgh Sleep Quality Index) across different application conditions (clip, pillow, diffuser, and control). Higher scores indicate poorer sleep quality. After controlling for baseline ratings, participants using a diffuser reported better sleep quality and less daytime tiredness than the control group in Phase 1 (*p < 0.05).

For application, a group difference was found in PSQI Total score (*F* [3,124] = 5.47, *p* = 0.001, partial η^2^ = 0.12), post hoc analysis suggested that participants using a diffuser had better subjective sleep quality than the pillow group (*p* < 0.001). There was a trend toward a significant group difference in subjective sleep quality (a component of PSQI, *F* [3,124] = 2.53, *p* = 0.06, partial η^2^ = 0.058), where participants using a diffuser had better sleep quality than those in the control group (*p* = 0.049, Fig. 3A). Moreover, the component of day tiredness was different (a component of PSQI, *F* [3,124] = 3.14, *p* = 0.028, partial η^2^ = 0.071, Fig. 3B), suggesting that, after controlling for baseline ratings, participants using a diffuser tended to feel less day tiredness than those in the control group (*p* = 0.052). No differences were found in other measures, nor for the Fitbit data (all *p* > 0.05).

### Linear mixed model results

For the PSQI total score, there was a trend-like interaction between session and application (*F* [4, 229] = 2.37, *p* = 0.054), with participants in the pillow group showing worse sleep quality in both phase 1 and phase 2 compared to baseline (*p* < 0.001 and *p* = 0.004, respectively), while no such effects were seen in other application groups. Diffuser group participants had better sleep quality than pillow group participants in phase 1 (*p* = 0.005). No significant interaction between odor and session or changes in odor thresholds (all *p* > 0.05). For Fitbit data, a main effect of odor was found for REM sleep time (*F* [2, 204] = 3.37, *p* = 0.036), with the “perfume” group tending to have more REM sleep than the lavender group (*p* = 0.092). Odor type also affected light sleep time (*F* [2, 186] = 4.42, *p* = 0.013), with lavender associated with more light sleep than orange (*p* = 0.023). No other significant results emerged (all *p* > 0.05).

## Discussion

The present study found that nighttime odor exposure can affect sleep. During the phase of odor exposure, participants exposed to the specially designed “perfume” reported better subjective sleep quality than those in the control group, after covarying for baseline ratings. Among the application methods tested, the use of a diffuser was associated with better sleep quality and reduced daytime tiredness compared to the control group, as reflected in the PSQI components. However, no significant differences were observed in objective sleep measures recorded by Fitbit devices, nor was there evidence of a sustained effect of odor on sleep quality over time.

Participants exposed to the “perfume” reported better subjective sleep quality, a component of the PSQI, compared to the control group, suggesting that the odor positively influenced sleep quality^5,9,11,18^. Essential oil inhalation has been shown to have calming and relaxant effects^19–21^, which may help individuals relax at night and thus improve sleep quality subjectively. However, in the current study, no differences of objective sleep quality as measured by the Fitbit were observed after odor exposure. One possible explanation is the limitation of the Fitbit device, which relies on accelerometry rather than, for example, electroencephalography. As a result, small or modest changes in sleep architecture may have gone undetected. One important point to note is that the current results indicate that odor exposure did not lead to sustained improvements in sleep quality over time, no matter subjectively or objectively, which is not align with previous findings^12^. Our results suggest only that during odor exposure, participants in the “perfume” and diffuser groups reported better subjective sleep quality, the component of the PSQI, than non-exposed controls. Future studies investigating home-based sleep quality should consider using more precise measures, such as EEG-based assessments^22^.

Nevertheless, the subtle to moderate effect of odor on sleep appears to be present, though it is likely driven more by indirect influences on mood and relaxation^19–21^. These effects seem to be modulated by the odor’s pleasantness and contextual factors, which may help explain the variability in reports on odors and sleep quality^3,23,24^.

Previous studies have primarily focused on the effects of lavender fragrance on sleep in rodents and human being^9,18,25^. For instance, in a randomized controlled trial in 67 participants, Chien and colleagues suggested inhaling lavender oil via a diffuser significantly improved sleep quality compared to the control group who received no odor^26^. Similarly, a single-blinded, randomized pilot study with 10 volunteers indicated a trend towards sleep improvement with lavender oil compared to almond oil^27^. Interestingly, however, in the present study, we did not observe a significant effect of lavender compared to the control condition but did find an effect with the “perfume”. This suggests that the impact of odors on sleep quality may be more general rather than specific to particular scents. It also argues against possible specific pharmacologic effects of individual odorants in terms of “perfume”. While the observed effect of the “perfume” may appear to relate to its complexity, it is important to note that other odors used in the study, such as lavender, are also composed of multiple compounds, e.g. linalool, linalyl acetate, acetic acid, limonene, or terpinen-4-ol^28^. Therefore, the effect is not likely to be solely attributable to the complexity of the odor.

Interestingly, the use of a diffuser was associated with better subjective sleep quality and reduced daytime tiredness, as reflected in the PSQI components, compared to the control group. These findings suggest that a diffuser may be a suitable method for delivering odors and enhancing the ambient sleep environment. Previous studies have suggested that different methods in aromatherapy can significantly influence the outcomes of sleep quality^11^. Unlike nasal clips and pillow applications, the diffuser provides a non-invasive method for presenting odors that is consistent with the non-arousing nature of selective olfactory stimuli during sleep^29^.

There are several limitations to the current study. First, the relatively small number of participants within each odor–application subgroup may have reduced statistical power and limited the ability to detect interaction effects. Future studies should include larger sample sizes to validate and extend our findings. Additionally, although we employed Fitbit devices as an objective measure of sleep, their limited sensitivity compared to polysomnography may have contributed to the absence of significant findings in the objective data. Another limitation is that participants’ personal experiences with odors, such as habitual perfume use, were not assessed, which may have influenced individual responses. Future studies should consider including such measures to better account for individual variability.

## Conclusion

The present results indicate that exposure to the specially designed “perfume” was associated with better subjective sleep quality compared to non-exposed controls. Using a diffuser to deliver the odor appears to be an effective method for creating a non-arousing environment. However, no sustained improvement in sleep quality over time was observed. Future studies should consider using more sensitive objective measures of sleep quality.

## Supplementary Information

Below is the link to the electronic supplementary material.

Supplementary Material 1

## Data Availability

The datasets generated during and/or analyzed during the current study are available from the corresponding author on reasonable request.
