# Supplementary material for: Effects of nighttime odor exposure and delivery methods on subjective sleep quality in healthy adults
Source: Sci Rep. 2025 Sep 12;15:32452. doi: 10.1038/s41598-025-18075-x (PMC12432149; doi:10.1038/s41598-025-18075-x)
Supplement: Supplementary file 1 — Supplementary Material 1 [file 41598_2025_18075_MOESM1_ESM.docx]

Pilot Study:

Two essential demands were made regarding the scents: Firstly, they should achieve a comparable level of scent intensity among themselves and across all application methods. Secondly, it was required that the fragrance remains perceptible at the end of each night – meaning that the participants were scented throughout the entire night.

Thus, prior to the study, it was necessary to determine the appropriate amount and concentration of fragrance for each of the nine groups. To achieve this, we conducted a pilot study before the main study, which consisted of two phases:

- Phase 1 of the Pilot Study: Laboratory Test Series

Primary objective: to roughly dose scent intensity comparably

Since the three pure fragrance oils exhibited different intensities, the first phase aimed to find a rough, comparable concentration. In the laboratory, a test series was conducted for all fragrances and their applicators. From this, concentrations were derived that appeared comparably strong under laboratory conditions.

To enable a subjective comparison of the individual application methods, the subjective perception of intensity was illustrated using a scale. The scale included statements such as "not at all", "barely perceptible", "moderately noticeable", "noticeable", "distinct", "strong" - these assignments were divided into numerical values corresponding to this order from 0-5. Since the scent intensity should be "distinct", the target of this pilot study was a numerical value of 4.

As a first approximation, the following preliminary concentrations for the individual groups resulted from this conducted test series:

| **Group** | **Concentration in Laboratory Phase** |
| --- | --- |
| **Clips Lavender** | 1/30 |
| **Clips Orange** | 1/40 |
| **Clips Odor X** | 1/50 |
| **Pillow Lavender** | 1/50 |
| **Pillow Orange** | 1/50 |
| **Pillow Odor X** | 1/50 |
| **Diffuser Lavender** | 1/20 |
| **Diffuser Orange** | 1/10 |
| **Diffuser Odor X** | 1/10 |

Originally, the goal was to scent the pillows with a spray. Since the intensity of these sprays could not be adequately determined in the laboratory, the preliminary concentration was set at 1/50.

(It turned out that Odor X was stronger in fragrance than Lavender and Orange. )

- Phase 2 of the Pilot Study: Field Test

Special emphasis: Scent longevity

Once comparable concentrations were found in the laboratory, it was necessary to practically test the scent duration and distribution in a sleep environment. The subjective intensity was set at a numerical value of 4, meaning "distinct", for the comparable beginning of each night. To ensure that all applicators emitted their scent throughout the entire night, the minimum durability was set at 8 hours.

For the application methods Clips and Diffuser, the following individual dosages were determined:

| **Gruppe** | **Konzentration Laborphase** |
| --- | --- |
| **Clips Lavender (each)** | 1/12 (0.25 ml Odorant to 2.75 ml Propylenglycol) |
| **Clips Orange (each)** | 1/20 (0.15 ml Odorant to 2.85 ml Propylenglycol) |
| **Clips Odor X (each)** | 1/40 (0.075 ml Odorant to 2.9 ml Propylenglycol) |
| **Pillow Lavender** | Pure |
| **Pillow Orange** | Pure |
| **Pillow Odor X** | Pure |
| **Diffuser Lavender** | 1/3 20 ml (6.7 ml Odorant to 13.3 ml Propylenglycol) |
| **Diffuser Orange** | 1/4 20 ml (5 ml Odorant to 15 ml Propylenglycol) |
| **Diffuser Odor X** | 1/7 20 ml (2.7 ml Odorant to 17.3 ml Propylenglycol) |

The most feasible total volume of the fragrance-solvent mixture was 20 ml for all three Diffuser groups.

In this test phase, we moved away from the idea of spraying the pillows with a fragrance-solvent mixture, as this would have led to the greasing of the pillowcase with the solvents in question and thus to skin contact with the mixture during sleep. Therefore, we switched to achieving the scent of the pillow group by spreading the pure fragrance oil onto the pillow. Here, the oil should be applied to the pillow so frequently that the scent intensity at the beginning of the night is distinctly perceptible (corresponding to scale 0-5, a value of 4).

As throughout the study, participants repeatedly rejected CL due to its too intense odor, we felt compelled to reduce the concentration of Clips from 1/8 to 1/12. Thus, in the CL group, there were 2 participants with a concentration of 1/8 and 7 participants with 1/12.
